# Identification of key genes and signaling pathways related to Hetian sheep wool density by RNA-seq technology

**DOI:** 10.1371/journal.pone.0265989

**Published:** 2022-05-25

**Authors:** Ruijun Shi, Shuwei Li, Penggang Liu, Shuhui Zhang, Zhenhui Wu, Tinghui Wu, Shujuan Gong, Yu Wan

**Affiliations:** 1 Key Laboratory of Protection & Utilization of Biological Resources in Tarim Basin, College of Life Sciences, Tarim University, Alar, China; 2 College of Veterinary Medicine, Yangzhou University, Yangzhou, China; 3 College of Animal Science and Veterinary Medicine, Shandong Agricultural University, Taian, China; University of Naples Federico II, ITALY

## Abstract

Hetian sheep is a breed of sheep unique to the Hetian area of Xinjiang whose wool is used for producing blankets. Individual differences and hair follicle density are the key factors affecting wool production. Therefore, this study aimed to assess the Hetian sheep having different wool densities to statistically analyze the wool traits and hair follicle parameters. Furthermore, the transcriptome sequencing analysis was performed on the skins with different wool densities. The results showed that wool quantity and total hair follicle density of the high wool density sheep was significantly higher than low wool density sheep. The sheepskin with high wool density was found to grow more and finer wool than sheepskin with low wool density. A total of 1,452 differentially expressed genes were screened from the two sets of samples, including 754 upregulated and 698 downregulated genes. The differentially expressed genes were involved in the TGF-β/BMP and MAPK signaling pathways related to hair growth. Eleven differentially expressed genes belonging to the KAPs and KIFs might affect the fineness of the wool. The key genes, like the *TNF*, *MAP2K2*, *INHBA*, *FST*, *PTPN11*, *MAP3K7*, *KIT*, and *BMPR1A*, were found to probably affect the growth and density of the wool. The qPCR verified eight genes related to the MAPK pathway whose gene expression trends were consistent with the transcriptome sequencing results. This study furnishes valuable resources for enhancing the quality and production of wool in the Hetian sheep.

## Introduction

Hetian sheep is a breed of wool-producing sheep that lives in the Hetian area of Xinjiang. It has the characteristics of heat resistance, roughage tolerance, and strong disease resistance. The Ministry of Agriculture has included Hetian sheep in the "National Protection List of Livestock and Poultry Genetic Resources." Hetian wool has long length, strong elasticity, high whiteness, and good bending resistance [1]. The Hetian carpet with a long history is made of Hetian wool. The carpet is famous globally because it has bright color, and a luxuriant feeling, and the carpet is comfortable, strong, and durable. The two types of hair follicles that support the growth of wool include primary and secondary follicles. Hair follicles are arranged regularly and continuously in the skin according to skin regions to form hair follicle groups, hair follicles occupy their respective positions in the hair follicle group, these groups determine the distribution of various types of wool in the skin [2]. Hair follicles are formed by the interaction between the embryonic neuroectoderm and the mesenchyme, they synthesize and secrete a variety of specific keratins and have a unique physiological structure, strong regenerative ability, and complex skin appendages with periodic growth characteristics [3]. Hair follicles run through the epidermis and dermis of mammalian skin. Hair follicles continue to undergo changes in anagen, catagen, and telogen phases, each phase is strictly regulated by genetic factors and is characterized by obvious changes in gene expression, cell proliferation, and cell differentiation [3, 4]. Many factors, such as heredity, nutrition, and environment, can affect the quality and yield of the coat of fur animals [5].

Hetian wool is composed of fine wool and coarse wool. Wool density between individual adult Hetian sheep differs, individuals with rich fine wool grow denser wool fibers, whereas sheep with rich coarse wool fibers have less wool. Most of the wool fiber is composed of a protein called α-keratin [6]. The main components of wool can be divided into two extractable proteins according to sulfur content, namely, S-carboxymethyl keratin A (SCMK-A) and S-carboxymethyl keratin B (SCMK-B), SCMK-A is wool KIF and SCMK-B is a wool KAP [7]. In the process of wool fiber growth, KAPs interact with KIFs, are cross-linked through disulfide bonds and combined by covalent bonds, form wool fibers through intermolecular or intramolecular disulfide bonds eventually [8, 9]. Hair production and hair follicle growth is a very complex process involving the regulation of numerous genes and a variety of complex signaling pathway networks [10–14]. The growth of the animal hair is affected by the signaling pathway through the delivery of ligands, receptor complexes, intermediate signal molecules, and downstream transcription factors, as well as their corresponding target gene genetic mutation mutations, epigenetic modifications, and post-translational protein modifications [15, 16]. The precise regulation of the activation time, strength, and duration of the signal pathway is also crucial for hair follicle morphogenesis and hair maintenance [17–19]. To understand the regulatory mechanism of animal hair growth in a better way studies should focus on the gene complexity and the multiple functions of the signaling pathways. There are only a few studies on the difference in wool density to date. The key genes and regulatory factors regulating wool density and growth remain elusive. The RNA sequencing (RNA-seq) technology has become a common tool to effectively analyze the periodic growth of skin hair follicles and the regulation mechanism of hair growth at the molecular biology level [20–22].

In this study, we mainly performed measured and statistically analyzed of wool traits and hair follicle parameters of Hetian sheep with different wool densities. Secondly, performed RNA-seq analysis for the skin with different wool densities of Hetian sheep to identify the genes and signal pathways related to wool growth and wool density. This research provides knowledge for exploring Hetian sheep wool output and improving wool production efficiency.

## Materials and methods

### Experimental design

The experimental sheep were selected from the flock of the Hetian sheep breeding base in Luopu County. The sheep of 10 high wool density and 10 low wool density were used as experimental animals, and their age (2 years) and sex (female) were consistent. All test sheep were raised under the same natural light cycle and natural temperature conditions. All procedures for animal testing are carried out in accordance with the Guidelines for the Care and Use of Research Animals in China (GB14925-2001). All trial protocol were approved by the Experimental Animal Center of Tarim University. The wool density at 10 cm behind the scapula was measured by wool stemple method (Agricultural Industry Standard of the People’s Republic of China, NY/T 1817–2009).

The 1 cm^2^ of wool was collected five times for each sheep, collected in zip locks bags were designed to measure the wool traits. The wool density of each sheep was recorded as the average of the five measurements using the following method: The wool bundle sample is required to be neat and complete. Cut a 1 cm wool bundle as the measuring wool sample, carefully put the wool sample into a 30 mL ×60 mL weighing bottle, and wash it with petroleum ether with a boiling point of 90°C~120°C. Weigh the total mass of the wool sample to be tested (K mg). Take a small bunch of wool (not less than 300 roots) from each weighed wool sample, and measure the total number (N_1_ roots) and total mass (K_1_ mg) of the wool. Then, the formula for calculating the number of wool (N roots) with a skin area of 1 cm^2^ is as follows: N = (K÷K_1_)×N_1_.

Ten sheep with high wool density and ten sheep with low wool density were divided into two groups, HS means sheep in the high wool density group, and HP means sheep in the low wool density group. We selected three sheep each from HS group and HP group. The wool at 10 cm from the posterior edge of the scapula was shaved, and the skin was disinfected and injected with local anesthetics. Samples were collected using a skin punch with a diameter of 1 cm. The samples were placed into the RNase-free cryotubes and quickly frozen in liquid nitrogen.

### Analysis of the wool traits

The wool samples of HS group and HP group were divided into two fiber types with fine wool and coarse wool. The wool fiber diameter of each sheep is measured. Randomly selecting 10 from the fine wool and randomly selecting 10 from the coarse wool. The diameter of the wool fiber was measured at a magnification of 100 times using a length measuring tool of the microscopic imaging system (DS-Fi3 Nikon Japan) using an optical microscope (Eclipse E200MV Nikon Japan). The morphology of the wool fiber was examined under a scanning electron microscope (EVO18 Zeiss Germany).

### Morphological examination of the skin

The skin samples were fixed for 48 h, dehydrated in a series of gradient alcohol concentrations, treated with xylene, and placed in paraffin at 65°C for 8 h. The skin samples were embedded with paraffin and were serially sectioned (cross-section; 6 μm) using a microtome (RM2016 Leica, Germany). The sections were subsequently placed on glass slides and incubated at 55°C for 5 h. The paraffin sections were subjected to the hematoxylin-eosin (H&E) staining (following the conventional procedure) and all the images were captured using an optical microscope (Eclipse E200MV Nikon Japan) and a microscopic imaging system (DS-Fi3 Nikon Japan). The parameters of the hair follicle were determined for six sheep, and three skin slices were analyzed from each sheep. For each section, ten different microscopic fields were randomly selected from the cross-section with the sebaceous gland layer, each having an area of 1 mm^2^. The numbers of primary hair follicles and secondary hair follicles were counted in each field of view and each data is the average of the number of hair follicles counted on a section under ten different microscopic fields [23].

### Transcriptome sequencing

TRIzol reagent (Invitrogen, CA, USA) was used according to the instructions to extract total RNA from the skin samples of the HS group (designated HS01, HS02, HS03) and HP group (designated HP01, HP02, and HP03). mRNA fragment was purified and reverse transcribed into cDNA. The adaptor was connected to the end of the double-stranded cDNA. The Illumina TruSeq RNA sample preparation kit (Illumina, San Diego, USA) was used to create a cDNA library by polymerase chain reaction (PCR). Six independent libraries were sequenced on the Illumina HiSeq 2500 instrument (ANOROAD, Beijing, China), according to the manufacturer’s instructions.

### Data analysis

FastQC was used to check the qality of the original RNA sequencing reads. The data was processed to remove impurities and obtain clean reads for subsequent bioinformatics analysis. The sieved out clean reads were mapped to the *Ovis aries* genome (*Oar_v3*.*1*). The raw count of each gene was determined by the default generalized linear model. Differential gene screening was performed using the factor of difference (fold change value) and q-value (adjusted p-value, p-value after correction) as related indicators. The genes with |log2(fold change)|>0.585 and q<0.05 were considered to be differentially expressed genes (DEGs).

All the DEGs were mapped to the Gene Ontology (GO) database (http://www.geneontology.org/) with *p* ≤ 0.05 as the threshold to define the GO functional classification with significant DEG enrichment. We used public pathway data (http://www.kegg.jp/) to conduct Kyoto Encyclopedia of Genes and Genomes (KEGG) analysis on the DEGs with *p* ≤ 0.05 as the threshold to define the pathways with significant DEG enrichment. Lianchuan Biological Cloud Platform (https://www.omicstudio.cn/index) was used for GO and KEGG visualization. Search tool for the Retrieval of Interacting Genes/Proteins STRING database (https://www.string-db.org/) was used in the protein–protein interaction (PPI) analysis of the DEGs. Cytoscape 3.8 was used for producing the PPI network maps and the hub clustering modules of the PPI network were further analyzed [24].

### Quantitative real-time PCR

The relative expression levels of eight DEGs screened from the DEG list were evaluated by qPCR to verify the RNA-seq results. The primer information is listed in S1 Table. Total RNA was extracted from the six skin tissues by TRIzol reagent (Invitrogen, CA, USA), and cDNA was synthesized by Reverse transcription kit (Transgen, Beijing, China). qPCR was performed in a BYQ6619-651598 instrument (Bioer Technology, Hangzhou, China) using TransStart Tip Green qPCR SuperMix (Transgen, Beijing, China). *GAPDH* as the internal reference gene. Amplification program: pre-denaturation at 94°C for 30 s, denaturation at 94°C for 5 s, and extension at 60°C for 30 s (40 cycles). qPCR data were generated from six independent samples (n = 6). The 2^−ΔΔCt^ algorithm was used to calculate the qPCR results. GraphPad Prism 8 software was used to make histograms.

### Statistical analyses

The statistical data of the two groups were compared through an independent sample *t*-test using the SPSS 23 software (SPSS Inc., Chicago, IL, USA). All the results have been expressed statistically as mean ± SD (standard deviation). *P* < 0.05 was considered to be a significant difference.

## Results

### Characterization of wool traits

**Fig 1:**
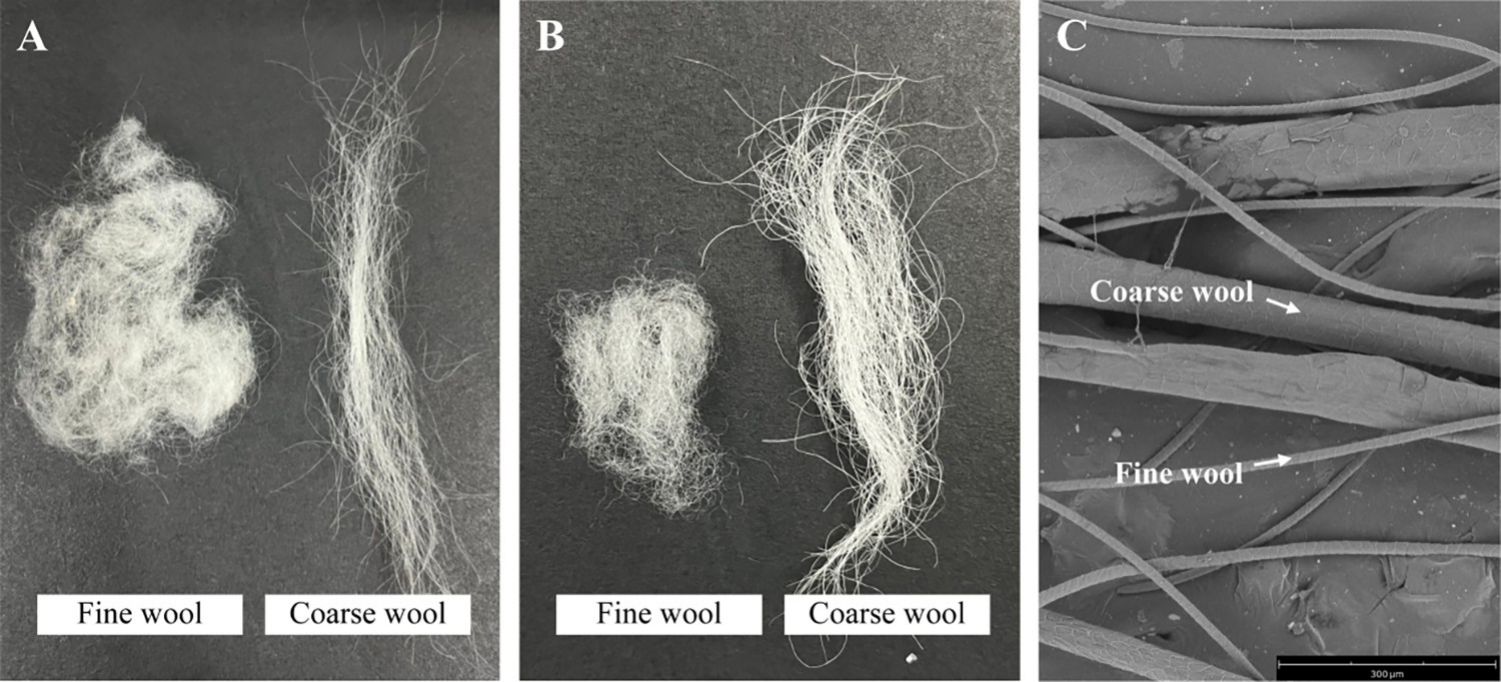
Wool characterization of the Hetian sheep based on the high and low wool density. (**A**) The wool samples of the HS. (**B**) The wool samples of the HP. (**C**) Microstructure of fine wool and coarse wool.

The wool of the Hetian sheep was observed to comprise fine wool and coarse wool. The wool with a diameter of fewer than 25 μm was defined as fine wool, while the wool with a diameter more than 25 μm was defined as coarse wool. The wool samples of the HS and HP groups were divided into two fiber types: fine wool and coarse wool (Fig 1A and 1B). When the microstructure of the fine wool and coarse wool was observed, the two kinds of wool fibers could be clearly distinguished under the scanning electron microscope (Fig 1C). Then, the wool traits of the HS and HP groups were measured and analyzed statistically (Table 1). The HS group demonstrated an average wool density of 1862.7 per cm^2^, while the HP group showed an average wool density of 1474.2 per cm^2^. The wool density of the HS group was found to be significantly higher than that of the HP group (*p* < 0.01). The ratio of the fine wool to coarse wool in the HS group was significantly higher than that in the HP group (*p* < 0.01). The average diameters of the fiber of coarse wool and fine wool in the HS group were 34.48 μm and 20.48 μm, respectively. On the other hand, the average diameters of the fiber of coarse wool and fine wool in the HP group were 40.96 μm and 21.4 μm, respectively. The diameters of the coarse wool fiber in the HS group were significantly higher than those in the HP group (*p* < 0.05). However, there was no significant difference in the diameter of the fine wool fiber.

**Table 1:** Comparison of the wool traits of the Hetian sheep.

Wool traits	Sheep of high wool density (n = 10)	Sheep of low wool density (n = 10)
The density of wool (per cm^2^)	1862.70±77.90**	1474.20±60.41
The ratio of fine wool to coarse wool	8.97±0.80**	4.12±0.38
The fiber diameter of the coarse wool (μm)	34.48±6.93	40.96±9.47*
The fiber diameter of the fine wool (μm)	20.48±2.88	21.40±2.47

**P < 0.01 and

*P < 0.05

### Morphological analysis of the skin

**Fig 2:**
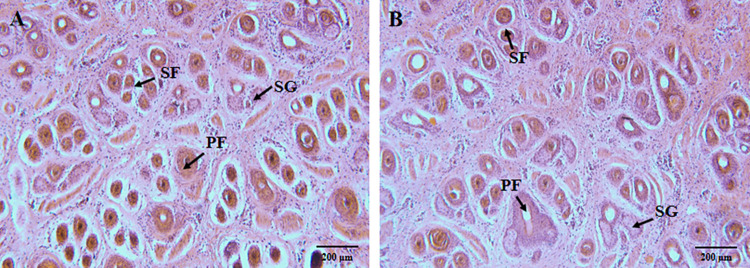
Skin morphology of the Hetian sheep with high and low wool density (100×). (A) Cross-sections of the sheepskin in the HS group. (B) Cross-sections of the sheepskin in the HP group. PF: primary hair follicle; SF: secondary hair follicle; SG: sebaceous gland.

The skin morphology of the Hetian sheep with different wool densities was observed. The H&E staining revealed the skin cross-section of the HS and HP groups (Fig 2A and 2B), with abundant hair follicles and sebaceous glands. The periphery of the primary hair follicles normally accompanied two sebaceous glands while the secondary hair follicles were found to be smaller in diameter than that of the primary hair follicles, and were clustered in distribution. The densities of the total hair follicle, primary hair follicles, and secondary hair follicles, as well as the ratio of the secondary to primary hair follicles between the two groups, were analyzed statistically (Table 2). The average total hair follicle density of the HS group was found to be 39.82 per mm^2^, and the average total hair follicle density of the HP group was found to be 31.95 per mm^2^. The hair follicle density of the HS group was found to be significantly higher than that of the HP group (*p* < 0.01). There was no difference in the density of the primary hair follicles between the two groups of skin. The average density of the secondary hair follicle of the HS and the HP groups were 33.79 per mm^2^ and 25.93 per mm^2^, respectively. While the density of the secondary hair follicle of the HS group was found to be significantly higher than that of the HP group (*p* < 0.01). The ratio of the secondary to primary hair follicles in the HS group was 5.64, the ratio of the secondary to primary hair follicles in the HP group was 4.23. The ratio in the HS group was significantly higher than that in the HP group (*p* < 0.01).

**Table 2:** Comparison of the parameters of the hair follicles of the Hetian sheep.

Hair follicle parameters	Sheep of high wool density (n = 3)	Sheep of low wool density (n = 3)
The total density of the hair follicles (per mm^2^)	39.82±2.40**	31.95±2.04
The density of the primary hair follicles (per mm^2^)	6.04±0.56	6.02±0.43
The density of the secondary hair follicles (per mm^2^)	33.79±2.30**	25.93±1.90
The ratio of the secondary hair follicles to primary hair follicles	5.64±0.64**	4.32±0.40

**P < 0.01 and

* P < 0.05

### Distribution statistics of the expression of the gene

**Fig 3:**
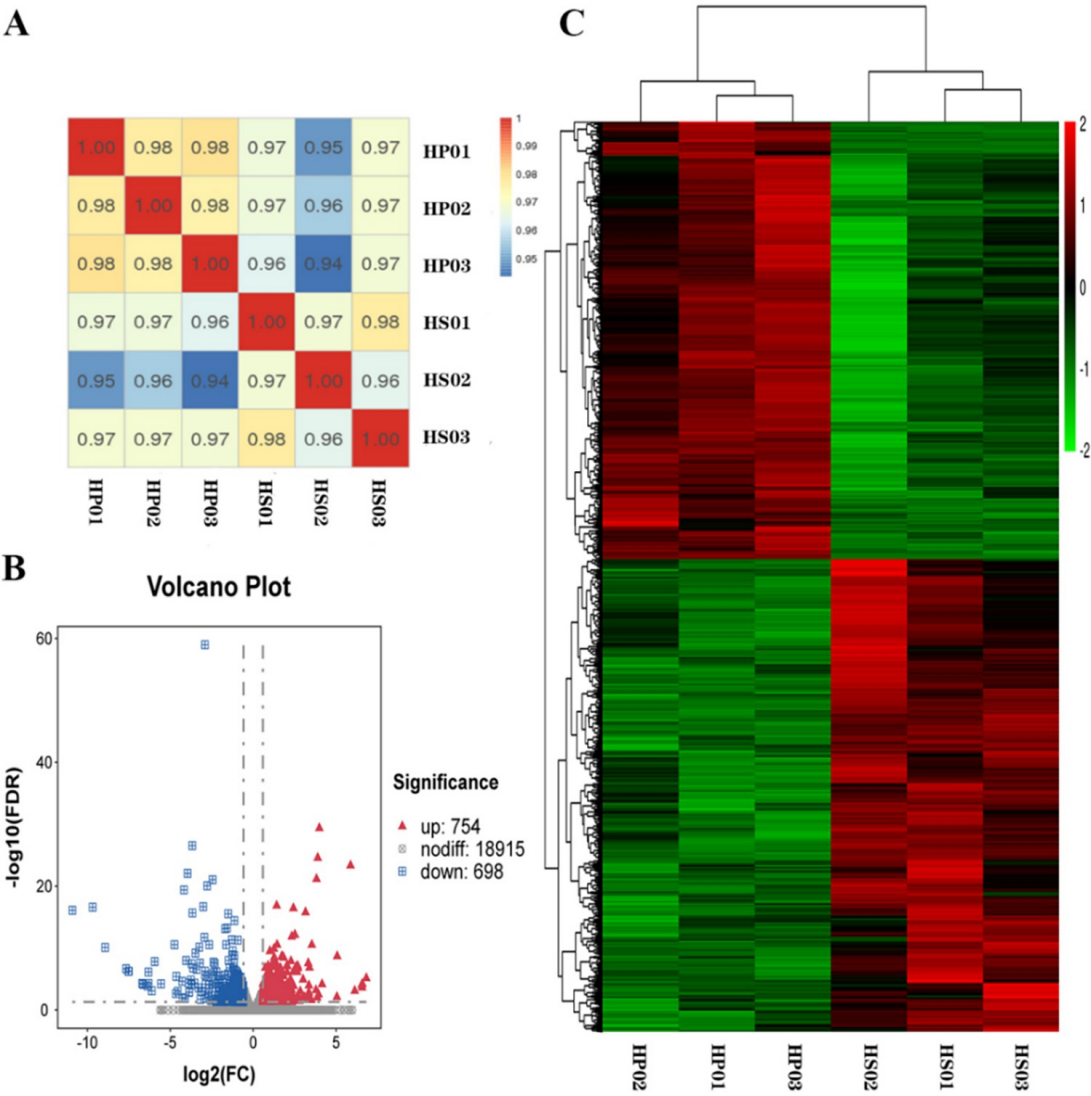
Preliminary analysis of the gene expression. (**A**) Heat map of gene expression correlation between samples. (**B**) Volcano plot of DEG screening. (**C**) Hierarchical clustering heat map of DEGs.

Six sample cDNA libraries were constructed to explore the molecular mechanism of the difference in wool density of Hetian sheep. Each library was sequenced and analyzed using Illumina HiSeq 2500, and the clean reads obtained were compared with the sheep reference genome (S2 Table). We calculated the correlation coefficient and studied the correlation to understand the overall similarity and difference of the gene expression patterns among all samples. Expectedly, all replicates showed high correlation in all comparisons (Fig 3A). Subsequently, we screened the DEGs with |log2(fold change)|>0.585 and q < 0.05. We found 20,367 transcripts that were jointly expressed by the two groups and 1,452 DEGs, including 754 upregulated and 698 downregulated genes, through the analysis of known coding genes (Fig 3B). A further hierarchical aggregation of DEGs was carried out. The skin samples of the three sheep in the HS group had similar gene expression patterns, which can be clearly distinguished from the expression patterns of the samples in the HP group (Fig 3C). The result indicates that the biological replicates have good reproducibility and the two groups of samples have differences in gene expression patterns.

### GO analysis of DEGs

**Fig 4:**
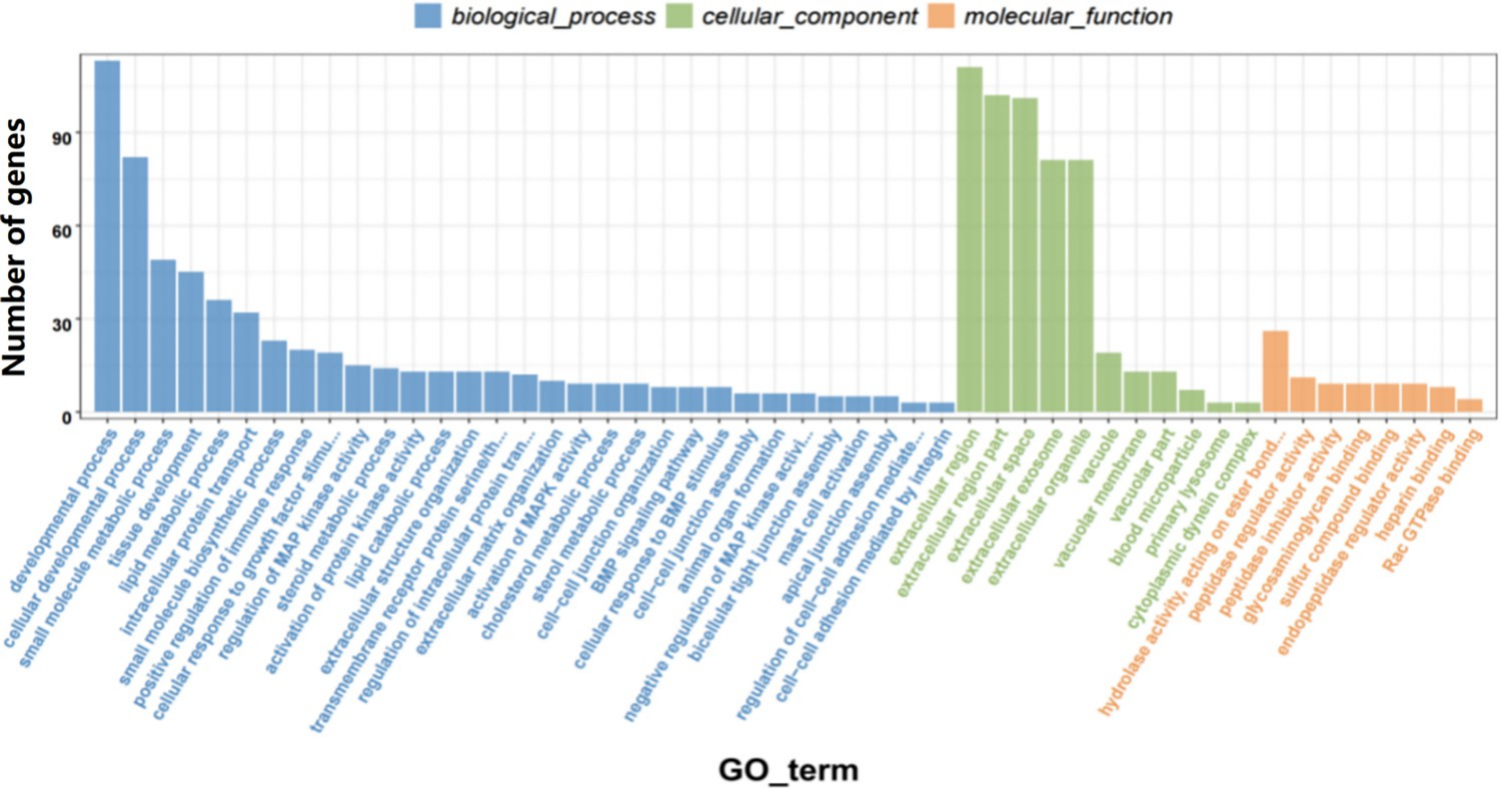
GO analysis of DEGs.

GO enrichment analysis revealed 1,452 DEGs were divided into 61 two-level categories. Biological processes accounted for 26 GO terms, molecular functions accounted for 14 GO terms, and cellular components accounted for 21 GO terms. The top 50 significantly enriched GO terms (*p* < 0.003) are shown in Fig 4. The functions of 82 genes belong to cellular developmental process, the functions of 45 genes belong to tissue development, the functions of 15 genes belong to the regulation of MAPK activity, and the functions of 13 genes belong to extracellular structure organization. In addition, the HS and HP groups had differences in BMP signaling pathway, bicellular tight junction assembly, extracellular matrix organization, activation of MAPK activity, negative regulation of MAPK activity, and bicellular tight junction assembly.

### KEGG analysis of DEGs

**Fig 5:**
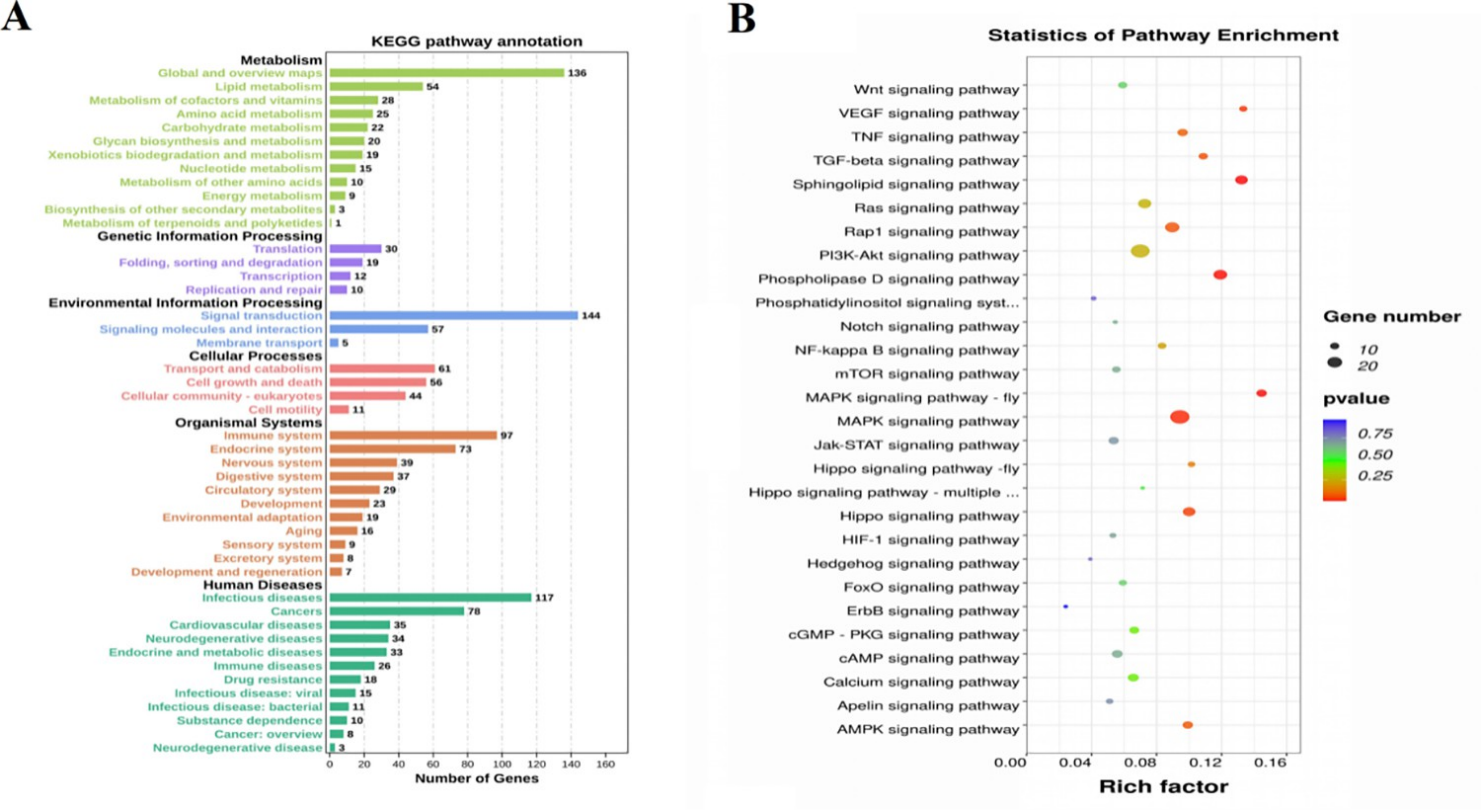
KEGG analysis of DEGs. (**A**) Primary and secondary KEGG classifications of DEGs. (**B**) Twenty-eight signaling pathways in environmental information processing. The circle size represents the number of genes and the color signifies *P*-value.

The 1,452 DEGs were divided into six primary KEGG categories (Fig 5A), namely, organic systems, metabolism, environmental information processing, cellular processing, genetic information processing, and human diseases. The organic systems category includes 10 secondary categories, metabolism includes 12 secondary categories, environmental information processing includes 3 secondary categories, cellular processing includes 4 secondary categories, and genetic information processing includes 4 secondary categories. The key signaling pathways that affect wool grow usually belong to signal transduction pathway in environmental information processing, which includes 28 signaling pathways (Fig 5B). MAPK signaling pathway-fly, Sphingolipid, phospholipase D, MAPK, VEGF, Hippo, and TGF-β signaling pathway were found to be significantly enriched (*p* < 0.05). Table 3 shows the corresponding up-regulated and down-regulated genes.

**Table 3:** The DEGs enriched the pathways related to environmental information processing.

Pathway	DEGs	
	Up	Down
Sphingolipid signaling pathway	*CTSD*, *FCER1G*, *MAP2K2*, *MAPK11*, *MAPK12*, *MAPK13*, *SMPD1*, *SPHK1*, *SPTLC1*, *TNF*	*ASAH1*, *FCER1A*, *MS4A2*, *PPP2R1B*, *PPP2R2B*, *PPP2R5E*
MAPK signaling pathway-fly	*DOK2*, *MAPK11*, *MAPK12*, *MAPK13*, *RASA3*	*IPO7*, *LOC101111026*, *MAP3K7*, *PTPN11*, *STRN*, *TBL1XR1*, *YWHAZ*
Phospholipase D signaling pathway	*AGPAT2*, *AGT*, *DGKQ*, *FCER1G*, *LPAR2*, *MAP2K2*, *SPHK1*, *TSC2*	*ADCY1*, *FCER1A*, *KIT*, *KITLG*, *LOC101120590*, *LPAR3*, *MS4A2*, *PTPN11*, *RRAS2*
MAPK signaling pathway	*AREG*, *CACNA1G*, *CACNB2*, *EFNA1*, *EFNA4*, *HSPA6*, *HSPB1*, *MAP2K2*, *MAPK11*, *MAPK12*, *MAPK13*, *MAPK7*, *MAPK8IP1*, *MAPKAPK2*, *NFKB2*, *RELB*, *TNF*, *VEGFB*	*CACNA1S*, *CHUK*, *DUSP4*, *FGF1*, *KIT*, *KITLG*, *MAP3K7*, *PLA2G4F*, *RPS6KA3*, *RRAS2*
VEGF signaling pathway	*HSPB1M*, *MAP2K2*, *MAPK11*, *MAPK12*, *MAPK13*, *MAPKAPK2*, *PLA2G4F*, *SPHK1*	
Hippo signaling pathway	*AREG*, *SCRIB*, *TP73*	*BMPR1A*, *BMPR2*, *CCND1*, *DLG1*, *FGF1*, *FRMD6*, *MOB1B*, *MPP5*, *PPP1CB*, *PPP2R1B*, *PPP2R2B*, *WNT2*, *YWHAZ*
TGF-β signaling pathway	*BMPR2*, *FST*, *TNF*	*BMPR1A*, *INHBA*, *PPP2R1B*, *RBL1*, *SMAD5*, *THSD4*, *ZFYVE16*

### PPI network analysis

**Fig 6:**
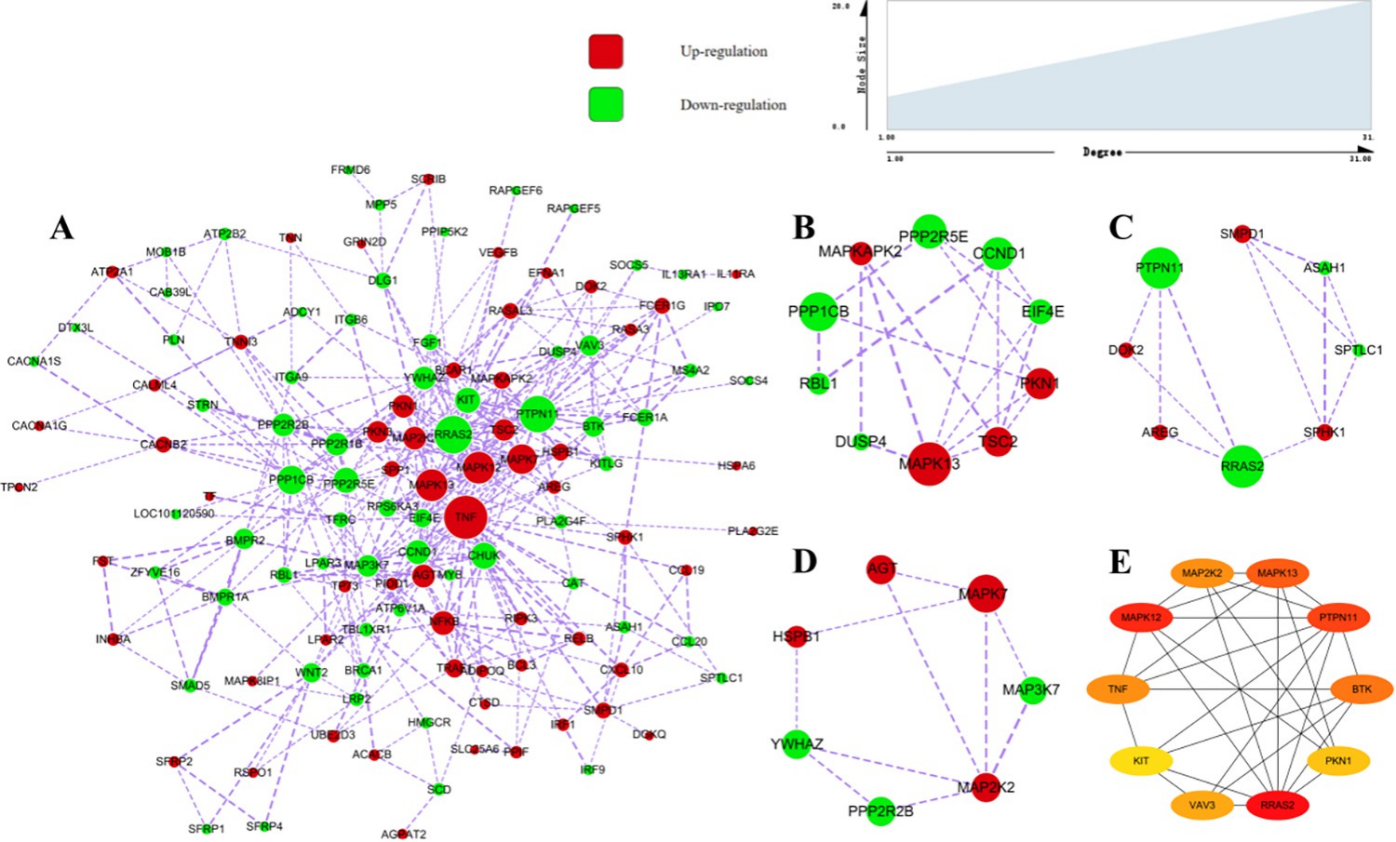
PPI network and hub clustering modules. **(A)** PPI network analysis for DEGs that were enriched in environmental information processing. **(B)** Cluster 1 (MCODE score = 4.0). **(C)** Cluster 2 (MCODE score = 3.4). **(D)** Cluster 3 (MCODE score = 3.3). (E) The interaction network of the top 10 genes. Red color represents the genes that were up-regulated and green color represents the down-regulated genes. The width of the edges has been related to the combined score between the two nodes. A wider edge indicates a larger combined score.

The PPI network analysis was performed for 169 DEGs that were enriched in twenty-eight signaling pathways in the environmental information processing (Fig 6). There were 123 proteins with existent interaction relationships and the size of each node was displayed according to the degree value (Fig 6A). A higher degree value indicated more biological processes to be involved. To identify other important clusters from the PPI network, a module analysis was conducted and 3 modules with the highest scores were selected. Cluster 1 contained 10 nodes and 18 interactions (Fig 6B). GO enrichment analysis suggested that genes in cluster 1 be mainly enriched in “protein serine phosphatase complex”, “myosin phosphatase activity”, “protein dephosphorylation”. Cluster 2 contained 8 nodes and 12 interactions (Fig 6C), where GO enrichment analysis suggested that genes in cluster 2 to be mainly enriched in “sphingomyelin metabolic process”, “ceramide biosynthetic process”, “sphingolipid biosynthetic process”. Cluster 3 contained 7 nodes and 10 interactions (Fig 6D), where the GO enrichment analysis suggested the genes in cluster 3 to be mainly enriched in “scaffold protein binding”, “MAP kinase activity”, “activation of MAPK activity”, “positive regulation of MAP kinase activity”. The 10 top nodes were identified by the degree value (Fig 6E). The highly expressed genes in the HS samples included *MAPK12*, *MAPK13*, *MAP2K2*, *TNF*, *PKN1*. The highly expressed genes in the HP samples included *RRAS2*, *PTPN11*, *BTK*, *VAV3*, *KIT*. GO enrichment analysis suggested 10 genes to be mainly were enriched in the “activation of MAPK activity”, “intracellular signal transduction”, “positive regulation of kinase activity”, “positive regulation of transferase activity”, “positive regulation of MAP kinase activity”. These identified genes might be partly related to the growth, and density of the wool (Fig 6B–6E).

### Differential expression of KAP and KIF genes in wool

GO enrichment analysis revealed that 14 genes belong to the functional categories of GO:0045095 (keratin filament) and GO:0045111 (intermediate filament). The expression levels of 11 genes (*LOC101116626*; *IFFO1*; *KRTAP12-1*; *LOC106990889*; *LOC105604748*; *KRTAP12-2*; *KRTAP16-1*; *KRT13*; *KRT15*; *KRT2*; *KRTAP1-3*) were higher in the HS group than in the HP group (Table 4).

**Table 4:** Expression of *KAP* and *KIF* genes.

Gene Name	Description	Log2(fold change (HS vs HP)	FDR
*LOC101116626*	*Ovis aries* keratin-associated protein 13-1-like	2.142418475	0.004709273
*IFFO1*	*Ovis aries* intermediate filament family orphan 1	0.944164489	0.012179038
*KRTAP12-1*	*Capra hircus* keratin associated protein 12.1	1.128151008	0.004388405
*LOC106990889*	*Ovis aries* keratin-associated protein 10-8-like	1.218460477	0.000974229
*LOC105604748*	*Ovis aries* musimon keratin-associated protein 10-3-like	0.789631794	0.025563337
*KRTAP12-2*	*Ovis aries* musimon keratin-associated protein 12–2	0.967095361	0.007342446
*KRTAP16-1*	*Ovis aries* musimon keratin-associated protein 16–1	0.730457832	0.016193473
*KRT38*	Capra hircus keratin, type I cuticular Ha7-like	−0.729614595	0.00211917
*KRT4*	*Ovis aries* keratin 4, type II (KRT4)	−2.650936545	0.0003354
*KRT13*	*Ovis aries* keratin, type I cytoskeletal 13	1.543337537	0.001134831
*KRT15*	*Ovis aries* keratin 15, type I	0.606452236	0.00758132
*KRT2*	Intermediate filament type II keratin	0.816893319	0.021112254
*GJA1*	*Ovis aries* gap junction protein alpha 1	−0.693112209	0.016759892
*KRTAP1-3*	Capra hircus keratin, high-sulfur matrix protein	1.197535581	0.0000293

### qPCR verification results

**Fig 7:**
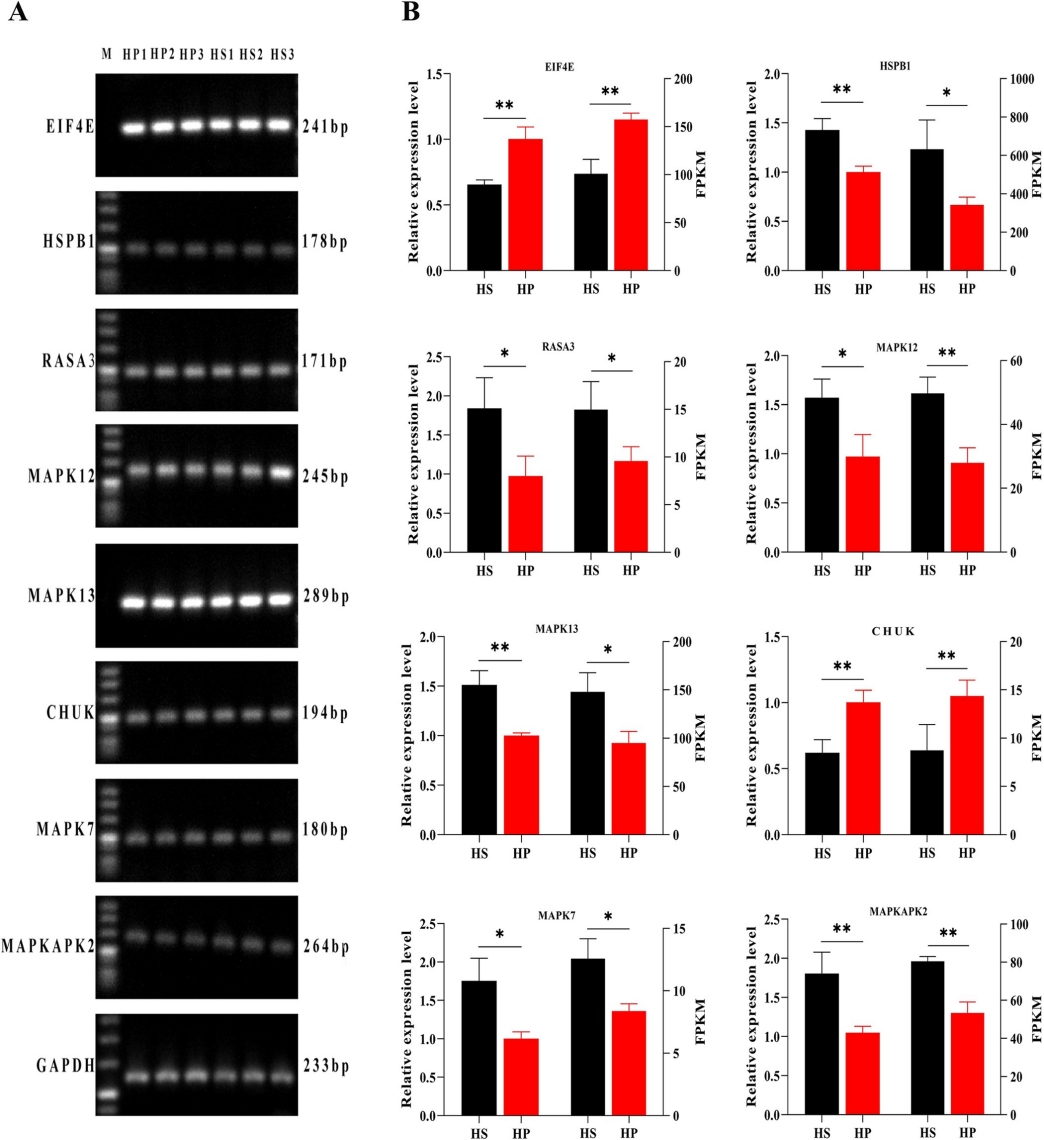
Eight genes selected for qPCR verification. (**A**) Electropherogram of the test genes and the internal reference gene (*GAPDH*). (**B**) Eight genes for qPCR detection and RNA-seq results. The left axis of the histogram represents the gene expression level, and the right axis of the histogram represents the FPKM value of the sequencing data. HS refers to the skin in the high wool density group, and HP refers to the skin in the low wool density group. (*p < 0.05 and **p < 0.01).

Eight genes related to the MAPK pathway were selected for qPCR verification to verify the reliability of the data. The internal reference gene was *GAPDH*. The results show that the gene expression data trends identified by relative quantification and RNA-seq are the same. The RNA-seq data is reliable, but the absolute expression levels from RNA-seq and qPCR are inconsistent (Fig 7). Six genes (*MAPK12*, *RASA3*, *MAPK7*, *MAPK13*, *HSPB1*, and *MAPK2*) were highly expressed in the HS group, and two genes (*CHUK* and *EIF4E*) were highly expressed in the HP group.

## Discussion

Wool-producing sheep breeds from Xinjiang were used as experimental animals, and the density of Hetian wool from sheep with similar body weights and ages under the same feeding conditions was quite different. According to research, the animals with slender hair fibers were found to have a higher density of the total hair follicle than the animals with coarse hair fibers, and the fiber diameter was negatively correlated with the density of the total hair follicle [25]. A similar observation was made in the Hetian sheep. On the whole, the sheep with high wool density were found to grow finer wool and the wool quality was found to be better than the sheep with low wool density. Moreover, the total hair follicle density and the ratio of the secondary to primary hair follicles of the high wool density skin were found to be higher than that of low wool density skin. Wool fiber is the external part of the hair follicle, and the quality of wool depends on the density of the hair follicle, as well as the type and size of the hair follicle [26]. From a genetic viewpoint, the main trait affecting the “quality” and “quantity” of the wool is the follicle density [27]. This study explored the difference in the wool traits of the Hetian sheep and compared the difference in the skin hair follicle density thereby providing a better analysis of the molecular determinants of these phenotypes.

Hair growth has a complex mechanism regulated by a variety of endogenous and exogenous factors [28]. Understanding the genetic basis of wool phenotype assist in improving the efficiency of wool production, and in identifying the molecular determinants of wool density and phenotype differences. At present, there are few to explore the genes and signaling pathways related to wool density, similar studies have been conducted mainly in rabbits [29–31]. Given this, the mRNA expression profile of the Hetian sheep skins with high and low wool densities was analyzed using RNA-seq technology, and the function of DEGs was searched to explain the molecular determinants of these differences in the wool and skin traits.

The GO analysis showed a large proportion of DEGs to be significantly enriched in the cellular developmental process, tissue development, regulation of MAPK activity, extracellular structure organization, bicellular tight junction assembly, and extracellular matrix organization. These DEGs might include the potential regulators of the density of the hair follicle and wool growth in the Hetian sheep. The KEGG enrichment analysis of the DEGs suggested seven significant enrichment pathways related to environmental information processing (Table 3). The MAPK and TGF-β signaling are known to function in regulating hair follicle morphogenesis and hair growth. The Sphingolipid, phospholipase D, VEGF, and Hippo signaling pathways might also have a role in wool growth and development.

MAPK plays an important role in cell proliferation, differentiation, growth, aging, and death [32, 33]. The MAPK signaling pathway regulates hair follicle circulation, wool growth, skin and hair follicle development, epidermal cell and keratinocyte differentiation, periodic wool growth, and wool fiber quality [34–37]. In addition, the upstream genes of the MAPK signaling pathway also play an important role in the regulation of the hair cycle and hair follicle stem cell self-renewal [38, 39]. In this study, most of the DEGs related to MAPK signaling are upregulated in skin with high hair density. These up-regulated genes might have a positive effect on the density and growth of wool. According to research, the TGF-β signaling maintains the epidermal and hair cycle homeostasis, regulating the proliferation, differentiation, and apoptosis of the different epithelial stem cell populations in the hair follicles [40]. Eight DEGs were found to be enriched in the BMP signaling pathway. BMPs serve as the regulator of vertebrate development and are known to have an important role in the morphogenesis and regeneration of hair follicles [41–43]. In addition, BMP signaling pathway signals can also control the hair follicle cycle by regulating the proliferation and differentiation of hair matrix precursor cells; it also has a regulatory effect on the size of hair follicles [44]. BMP signaling delays the development of hair follicles and may control the distance between follicles during embryogenesis [45]. Therefore, 15 DEGs in the TGF-β/BMP signaling pathway might affect the density and growth of the wool. Most genes in the TGF-β/BMP signaling pathway were found to express higher in the sheep having low wool density. The PPI network analysis was performed on the DEGs related to environmental information processing. The 10 top nodes were identified, the up-regulated genes in the HS samples included *MAPK12*, *MAPK13*, *MAP2K2*, *TNF*, *PKN1*, the up-regulated genes in the HP samples included *RRAS2*, *PTPN11*, *BTK*, *VAV3*, *KIT*. *TNF* participates in hair follicle circulation and delayed hair follicle entry into catagen phase [46, 47]. *TNF* plays an important role in wound-induced hair anagen growth and hair follicle neogenesis. In epidermal stem cells, *TNF* causes AKT phosphorylation and β-catenin activation [48]. Mutations in the *MAP2K2* gene can cause patients to show facial deformities, skin and hair abnormalities, physical dysplasia [49]. No reports have associated *MAPK12*, *MAPK13*, *PKN1*, *RRAS2*, *BTK* and *VAV3* to hair follicle and hair. These several genes are worthy of attention, and whether they play a role in regulating wool growth and regulating wool density requires further research. The *INHBA* and *FST* are up-regulated expressed in HS skin. *INHBA* is a member of the TGF-β superfamily, which plays a role in reproduction and development. Homozygous mouse lacking *INHBA* show dysfunctional beard, palate, and tooth development [50]. *FST* overexpression in sheep remarkably promotes the proliferation of sheep fetal fibroblasts and human keratinocytes [51]. In summary, the increased expression of *TNF*, *MAP2K2*, *INHBA*, and *FST* may have a positive effect on wool growth and wool density.

Mutations in the *PTPN11* gene can lead to the rough hair, sparse eyebrows, skin keratinization, and obvious hypertension [52]. A study on keratinocyte-specific *MAP3K7*-deficient mice showed that *MAP3K7* can regulate the growth, differentiation, and apoptosis of keratinocytes [53]. *MAP3K7* defects can remarkably decrease E15.5 fetal mouse hair follicle precursors and delay the morphogenesis of hair follicles; the loss of *MAP3K7* in hair follicles that are already in the anagen phase can lead to the passive apoptosis and degeneration of hair follicles and hair follicle damage [54]. Mutations in the *KIT* gene can cause a rare autosomal dominant melanocyte developmental disorder, which leads to skin and hair depigmentation and decreased hair density; patient with this disorder have thin hair, auburn hair, white hair [55, 56]. The destruction of the BMP receptor, *BMPR1A*, in mutant mouse leads to the severe impairment of the differentiation of the inner root sheath, the reduction of hair follicles in the dermis and subcutaneous tissues, and the decline of circulating epithelial cells; *BMPR1A* is necessary to complete tooth morphogenesis and regulate the terminal differentiation and proliferation of hair follicles after birth [57, 58]. In summary, *PTPN11*, *MAP3K7*, *KIT*, and *BMPR1A* genes may have an effect on the morphogenesis and wool growth of hair follicles, and they are all highly expressed in sheep skin with low wool density.

In addition, we found 14 DEGs belonging to KAPs and KIFs, eleven of which were up-regulated in high-wool density skin and three were up-regulated in low-wool density skin. We can come to the conclusion from the results of the wool phenotype of Hetian sheep with different wool densities, the Hetian sheep with high wool density grows more and finer wool, but the wool of low wool density Hetian sheep is thicker overall. In view of these findings, these 11 genes *(LOC101116626*, *IFFO1*, *KRTAP12-1*, *LOC106990889*, *LOC105604748*, *KRTAP12-2*, *KRTAP16-1*, *KRT13*, *KRT15*, *KRT2*, *KRTAP1-3*) may play a role in affecting the fineness of wool.

Finally, we verified the sequencing results by qPCR. The results showed that the sequencing results can correctly reflect the expression ability of the detected genes. The result shows that using this technology is an economical, rapid, efficient, and reliable method for the screening of DEGs in skin tissue. Analyzing gene expression at the genome level, exploring the internal connections, and studying the molecular biological mechanisms that lead to differences in wool traits are powerful tools for studying wool quality.

## Conclusions

In summary, this study has highlighted significant differences in the wool quantity and total hair follicle density in the Hetian sheep with high and low wool density. The skin with high wool density tends to grow more and finer wool than the skin with low wool density. It has detected the mRNA expression profile of the Hetian sheepskin with different wool densities and has screened out 1,452 DEGs. The DEGs involved in the TGF-β/BMP and MAPK signaling pathways related to hair growth. Eleven genes belonging to the KAPs and KIFs which might affect the fineness of the wool. The key genes, such as *TNF*, *MAP2K2*, *INHBA*, *FST*, *PTPN11*, *MAP3K7*, *KIT*, and *BMPR1A*, might affect wool growth and density. This study furnished valuable resources for enhancing the wool quality and quantity of the Hetian sheep.

## Supporting information

S1 Table qPCR primer sequence. (XLSX)

S2 Table Reference genome annotation statistics results of clean reads. (XLSX)

S3 Table 1,452 DEGs expression and annotation information. (XLSX)

S4 Table Signaling pathways in environmental information processing. (XLSX)

S1 Raw images (TIF)

## References

[1] Wang HY, Li SW, Wu TH, Wu ZH, Guo JX. The effect of androgen on wool follicles and keratin production in Hetian sheep. Braz J Biol. 2021;81(3):526–36. 10.1590/1519-6984.224056 https://search.crossref.org/?q=The+effect+of+androgen+on+wool+follicles+and+keratin+production+in+Hetian+sheep&from_ui=yes. 33470295

[2] Paus R, Cotsarelis G. The biology of hair follicles. N Engl J Med. 1999;341(7):491–7. 10.1056/nejm199908123410706 https://search.crossref.org/?q=The+biology+of+hair+follicles.+N+Engl+J+Med&from_ui=yes . 10441606

[3] Millar SE. Molecular mechanisms regulating hair follicle development. J Invest Dermatol. 2002;118(2):216–25. 10.1046/j.0022-202x.2001.01670.x https://search.crossref.org/?q=Molecular+mechanisms+regulating+hair+follicle+development&from_ui=yes . 11841536

[4] Schneider MR, Schmidt-Ullrich R, Paus R. The hair follicle as a dynamic miniorgan. Curr Biol. 2009;19(3):R132–42. 10.1016/j.cub.2008.12.005 https://search.crossref.org/?q=The+hair+follicle+as+a+dynamic+miniorgan&from_ui=yes . 19211055

[5] Muller-Rover S, Handjiski B, van der Veen C, Eichmuller S, Foitzik K, McKay IA, et al. A comprehensive guide for the accurate classification of murine hair follicles in distinct hair cycle stages. J Invest Dermatol. 2001;117(1):3–15. 10.1046/j.0022-202x.2001.01377.x https://search.crossref.org/?q=A+comprehensive+guide+for+the+accurate+classification+of+murine+hair+follicles+in+distinct+hair+cycle+stages&from_ui=yes . 11442744

[6] Popescu C, Hocker H. Hair—the most sophisticated biological composite material. Chem Soc Rev. 2007;36(8):1282–91. 10.1039/b604537p https://search.crossref.org/?q=Hair—the+most+sophisticated+biological+composite+material&from_ui=yes . 17619688

[7] Gong H, Zhou H, Forrest RH, Li S, Wang J, Dyer JM, et al. Wool Keratin-Associated Protein Genes in Sheep-A Review. Genes (Basel). 2016;7(6):24. 10.3390/genes7060024 https://search.crossref.org/?q=Wool+Keratin-Associated+Protein+Genes+in+Sheep-A+Review&from_ui=yes . 27240405 PMC4929423

[8] Powell BC, Rogers GE. The role of keratin proteins and their genes in the growth, structure and properties of hair. EXS. 1997;78:59–148. 10.1007/978-3-0348-9223-0_3 https://search.crossref.org/?q=The+role+of+keratin+proteins+and+their+genes+in+the+growth,+structure+and+properties+of+hair&from_ui=yes . 8962491

[9] Yuan S, Li F, Meng Q, Zhao Y, Chen L, Zhang H, et al. Post-transcriptional Regulation of Keratinocyte Progenitor Cell Expansion, Differentiation and Hair Follicle Regression by miR-22. PLoS Genet. 2015;11(5):e1005253. 10.1371/journal.pgen.1005253 https://search.crossref.org/?q=Post-transcriptional+Regulation+of+Keratinocyte+Progenitor+Cell+Expansion,+Differentiation+and+Hair+Follicle+Regression+by+miR-22&from_ui=yes . 26020521 PMC4447420

[10] Biggs LC, Mikkola ML. Early inductive events in ectodermal appendage morphogenesis. Semin Cell Dev Biol. 2014;25–26:11–21. 10.1016/j.semcdb.2014.01.007 https://search.crossref.org/?q=Early+inductive+events+in+ectodermal+appendage+morphogenesis&from_ui=yes . 24487243

[11] Sennett R, Rendl M. Mesenchymal-epithelial interactions during hair follicle morphogenesis and cycling. Semin Cell Dev Biol. 2012;23(8):917–27. 10.1016/j.semcdb.2012.08.011 . 22960356 PMC3496047

[12] Lien WH, Fuchs E. Wnt some lose some: transcriptional governance of stem cells by Wnt/beta-catenin signaling. Genes Dev. 2014;28(14):1517–32. 10.1101/gad.244772.114 https://search.crossref.org/?q=+Wnt+some+lose+some:+transcriptional+governance+of+stem+cells+by+Wnt/beta-catenin+signaling&from_ui=yes . 25030692 PMC4102759

[13] Li A, Lai YC, Figueroa S, Yang T, Widelitz RB, Kobielak K, et al. Deciphering principles of morphogenesis from temporal and spatial patterns on the integument. Dev Dyn. 2015;244(8):905–20. 10.1002/dvdy.24281 https://search.crossref.org/?q=+Deciphering+principles+of+morphogenesis+from+temporal+and+spatial+patterns+on+the+integument&from_ui=yes . 25858668 PMC4520785

[14] Cetera M, Leybova L, Joyce B, Devenport D. Counter-rotational cell flows drive morphological and cell fate asymmetries in mammalian hair follicles. Nat Cell Biol. 2018;20(5):541–52. 10.1038/s41556-018-0082-7 https://search.crossref.org/?q=+Counter-rotational+cell+flows+drive+morphological+and+cell+fate+asymmetries+in+mammalian+hair+follicles&from_ui=yes . 29662173 PMC6065250

[15] Rippa AL, Vorotelyak EA, Vasiliev AV, Terskikh VV. The role of integrins in the development and homeostasis of the epidermis and skin appendages. Acta Naturae. 2013;5(4):22–33. 10.32607/20758251-2013-5-4-22-33 https://search.crossref.org/?q=+The+role+of+integrins+in+the+development+and+homeostasis+of+the+epidermis+and+skin+appendages&from_ui=yes . 24455180 PMC3890986

[16] Sotiropoulou PA, Blanpain C. Development and homeostasis of the skin epidermis. Cold Spring Harb Perspect Biol. 2012;4(7):a008383. 10.1101/cshperspect.a008383 https://search.crossref.org/?q=+Development+and+homeostasis+of+the+skin+epidermis&from_ui=yes . 22751151 PMC3385954

[17] Lee J, Tumbar T. Hairy tale of signaling in hair follicle development and cycling. Semin Cell Dev Biol. 2012;23(8):906–16. 10.1016/j.semcdb.2012.08.003 https://search.crossref.org/?q=+Hairy+tale+of+signaling+in+hair+follicle+development+and+cycling&from_ui=yes . 22939761 PMC3496046

[18] Wang X, Yu H, Lei A, Zhou J, Zeng W, Zhu H, et al. Generation of gene-modified goats targeting MSTN and FGF5 via zygote injection of CRISPR/Cas9 system. Sci Rep. 2015;5:13878. 10.1038/srep13878 https://search.crossref.org/?q=+Generation+of+gene-modified+goats+targeting+MSTN+and+FGF5+via+zygote+injection+of+CRISPR/Cas9+system&from_ui=yes . 26354037 PMC4564737

[19] Qiao X, Wu JH, Wu RB, Su R, Li C, Zhang YJ, et al. Discovery of differentially expressed genes in cashmere goat (Capra hircus) hair follicles by RNA sequencing. Genet Mol Res. 2016;15(3). 10.4238/gmr.15038589 https://search.crossref.org/?q=+Discovery+of+differentially+expressed+genes+in+cashmere+goat+(Capra+hircus)+hair+follicles+by+RNA+sequencing&from_ui=yes . 27706691

[20] He N, Su R, Wang Z, Zhang Y, Li J. Exploring differentially expressed genes between anagen and telogen secondary hair follicle stem cells from the Cashmere goat (Capra hircus) by RNA-Seq. PLoS One. 2020;15(4):e0231376. 10.1371/journal. pone.0231376 https://search.crossref.org/?q=+Exploring+differentially+expressed+genes+between+anagen+and+telogen+secondary+hair+follicle+stem+cells+from+the+Cashmere+goat+(Capra+hircus)+by+RNA-Seq&from_ui=yes . 32298297 PMC7162518

[21] Lv X, Chen L, He S, Liu C, Han B, Liu Z, et al. Effect of Nutritional Restriction on the Hair Follicles Development and Skin Transcriptome of Chinese Merino Sheep. Animals (Basel). 2020;10(6):1058. 10.3390/ani10061058 https://search.crossref.org/?q=+Effect+of+Nutritional+Restriction+on+the+Hair+Follicles+Development+and+Skin+Transcriptome+of+Chinese+Merino+Sheep&from_ui=yes . 32575477 PMC7341508

[22] Wang J, Sui J, Mao C, Li X, Chen X, Liang C, et al. Identification of Key Pathways and Genes Related to the Development of Hair Follicle Cycle in Cashmere Goats. Genes (Basel). 2021;12(2):180. 10.3390/genes12020180 https://search.crossref.org/?q=+Identification+of+Key+Pathways+and+Genes+Related+to+the+Development+of+Hair+Follicle+Cycle+in+Cashmere+Goats&from_ui=yes . 33513983 PMC7911279

[23] Yang CH, Xu JH, Ren QC, Duan T, Mo F, Zhang W. Melatonin promotes secondary hair follicle development of early postnatal cashmere goat and improves cashmere quantity and quality by enhancing antioxidant capacity and suppressing apoptosis. J Pineal Res. 2019;67(1):e12569. 10.1111/jpi.12569 . 30861591

[24] Xin S, Zhang W. Construction and analysis of the protein-protein interaction network for the olfactory system of the silkworm Bombyx mori. Arch Insect Biochem Physiol. 2020;105(3):e21737. 10.1002/arch.21850 . 32926465

[25] Moore KE, Maloney SK, Blache D. High follicle density does not decrease sweat gland density in Huacaya alpacas. J Therm Biol. 2015;47:1–6. 10.1016/j.jtherbio.2014.10.009 https://search.crossref.org/?q=High+follicle+density+does+not+decrease+sweat+gland+density+in+Huacaya+alpacas&from_ui=yes . 25526647

[26] Li S, Chen W, Zheng X, Liu Z, Yang G, Hu X, et al. Comparative investigation of coarse and fine wool sheep skin indicates the early regulators for skin and wool diversity. Gene. 2020;758:144968. 10.1016/j.gene.2020.144968 . 32707304

[27] Doyle EK, Preston JWV, McGregor BA, Hynd PI. The science behind the wool industry. The importance and value of wool production from sheep. Anim Front. 2021;11(2):15–23. 10.1093/af/vfab005 . 34026311 PMC8127695

[28] Bhat B, Yaseen M, Singh A, Ahmad SM, Ganai NA. Identification of potential key genes and pathways associated with the Pashmina fiber initiation using RNA-Seq and integrated bioinformatics analysis. Sci Rep. 2021;11(1):1766. 10.1038/s41598-021-81471-6 . 33469142 PMC7815713

[29] Zhao J, Liu N, Liu K, He J, Yu J, Bu R, et al. Identification of genes and proteins associated with anagen wool growth. Anim Genet. 2017;48(1):67–79. 10.1111/age.12480 . 27611105

[30] Ding H, Zhao H, Zhao X, Qi Y, Wang X, Huang D. Analysis of histology and long noncoding RNAs involved in the rabbit hair follicle density using RNA sequencing. BMC Genomics. 2021;22(1):89. 10.21203/rs.3.rs-43570/v2 . 33509078 PMC7845105

[31] CHEN S-j, LIU T, LIU Y-j, DONG B, GU Z-l. Gene Expression Patterns in Different Wool Densities of Rex Rabbit Using cDNA Microarray. Agricultural Sciences in China. 2011;10(4):595–601. 10.1016/s1671-2927(11)60041-2.

[32] Tidyman WE, Rauen KA. The RASopathies: developmental syndromes of Ras/MAPK pathway dysregulation. Curr Opin Genet Dev. 2009;19(3):230–6. 10.1016/j.gde.2009.04.001 https://search.crossref.org/?q=The+RASopathies:+developmental+syndromes+of+Ras/MAPK+pathway+dysregulation.+Curr+Opin+Genet+Dev&from_ui=yes . 19467855 PMC2743116

[33] Hancock JF. Ras proteins: different signals from different locations. Nat Rev Mol Cell Biol. 2003;4(5):373–84. doi: 10.1038/nrm1105//doi.org/10.1038/nrm1105 https://search.crossref.org/?q=Ras+proteins:+different+signals+from+different+locations&from_ui=yes . 12728271

[34] Zhang Y, Wu K, Wang L, Wang Z, Han W, Chen D, et al. Comparative study on seasonal hair follicle cycling by analysis of the transcriptomes from cashmere and milk goats. Genomics. 2020;112(1):332–45. 10.1016/j.ygeno.2019.02.013 https://search.crossref.org/?q=Comparative+study+on+seasonal+hair+follicle+cycling+by+analysis+of+the+transcriptomes+from+cashmere+and+milk+goats&from_ui=yes . 30779940

[35] Guo H, Cheng G, Li Y, Zhang H, Qin K. A Screen for Key Genes and Pathways Involved in High-Quality Brush Hair in the Yangtze River Delta White Goat. PLoS One. 2017;12(1):e0169820. 10.1371/journal.pone.0169820 https://search.crossref.org/?q=A+Screen+for+Key+Genes+and+Pathways+Involved+in+High-Quality+Brush+Hair+in+the+Yangtze+River+Delta+White+Goat&from_ui=yes . 28125615 PMC5268778

[36] Zhao B, Chen Y, Yan X, Hao Y, Zhu J, Weng Q, et al. Gene expression profiling analysis reveals fur development in rex rabbits (Oryctolagus cuniculus). Genome. 2017;60(12):1060–7. 10.1139/gen-2017-0003 https://search.crossref.org/?q=Gene+expression+profiling+analysis+reveals+fur+development+in+rex+rabbits+(Oryctolagus+cuniculus)&from_ui=yes . 28850794

[37] Sulayman A, Tian K, Huang X, Tian Y, Xu X, Fu X, et al. Genome-wide identification and characterization of long non-coding RNAs expressed during sheep fetal and postnatal hair follicle development. Sci Rep. 2019;9(1):8501. 10.1038/s41598-019-44600-w https://search.crossref.org/?q=Genome-wide+identification+and+characterization+of+long+non-coding+RNAs+expressed+during+sheep+fetal+and+postnatal+hair+follicle+developmen&from_ui=yes . 31186438 PMC6559957

[38] Akilli Ozturk O, Pakula H, Chmielowiec J, Qi J, Stein S, Lan L, et al. Gab1 and Mapk Signaling Are Essential in the Hair Cycle and Hair Follicle Stem Cell Quiescence. Cell Rep. 2015;13(3):561–72. 10.1016/j.celrep.2015.09.015 https://search.crossref.org/?q=Gab1+and+Mapk+Signaling+Are+Essential+in+the+Hair+Cycle+and+Hair+Follicle+Stem+Cell+Quiescence&from_ui=yes . 26456821

[39] Lv X, Chen W, Sun W, Hussain Z, Wang S, Wang J. Analysis of lncRNAs Expression Profiles in Hair Follicle of Hu Sheep Lambskin. Animals (Basel). 2020;10(6):1035. 10.3390/ani10061035 https://search.crossref.org/?q=Analysis+of+lncRNAs+Expression+Profiles+in+Hair+Follicle+of+Hu+Sheep+Lambskin&from_ui=yes . 32549352 PMC7341247

[40] Lin HY, Yang LT. Differential response of epithelial stem cell populations in hair follicles to TGF-beta signaling. Dev Biol. 2013;373(2):394–406. 10.1016/j.ydbio.2012.10.021 https://search.crossref.org/?q=Differential+response+of+epithelial+stem+cell+populations+in+hair+follicles+to+TGF-beta+signaling.+Dev+Bio&from_ui=yes . 23103542

[41] Eixarch H, Calvo-Barreiro L, Costa C, Reverter-Vives G, Castillo M, Gil V, et al. Inhibition of the BMP Signaling Pathway Ameliorated Established Clinical Symptoms of Experimental Autoimmune Encephalomyelitis. Neurotherapeutics. 2020;17(4):1988–2003. 10.1007/s13311-020-00885-8 https://search.crossref.org/?q=Inhibition+of+the+BMP+Signaling+Pathway+Ameliorated+Established+Clinical+Symptoms+of+Experimental+Autoimmune+Encephalomyeliti&from_ui=yes . 32681355 PMC7851289

[42] Mishina Y. Function of bone morphogenetic protein signaling during mouse development. Front Biosci. 2003;8(4):d855–69. 10.2741/1097 . 12700086

[43] Mayer JA, Foley J, De La Cruz D, Chuong CM, Widelitz R. Conversion of the nipple to hair-bearing epithelia by lowering bone morphogenetic protein pathway activity at the dermal-epidermal interface. Am J Pathol. 2008;173(5):1339–48. 10.2353/ajpath.2008.070920 https://search.crossref.org/?q=Conversion+of+the+nipple+to+hair-bearing+epithelia+by+lowering+bone+morphogenetic+protein+pathway+activity+at+the+dermal-epidermal+interface&from_ui=yes . 18832580 PMC2570124

[44] Sharov AA, Sharova TY, Mardaryev AN, Tommasi di Vignano A, Atoyan R, Weiner L, et al. Bone morphogenetic protein signaling regulates the size of hair follicles and modulates the expression of cell cycle-associated genes. Proc Natl Acad Sci U S A. 2006;103(48):18166–71. 10.1073/pnas.0608899103 https://search.crossref.org/?q=Bone+morphogenetic+protein+signaling+regulates+the+size+of+hair+follicles+and+modulates+the+expression+of+cell+cycle-associated+genes&from_ui=yes . 17114283 PMC1838724

[45] Botchkarev VA, Botchkareva NV, Roth W, Nakamura M, Chen LH, Herzog W, et al. Noggin is a mesenchymally derived stimulator of hair-follicle induction. Nat Cell Biol. 1999;1(3):158–64. 10.1038/11078 https://search.crossref.org/?q=Noggin+is+a+mesenchymally+derived+stimulator+of+hair-follicle+induction&from_ui=yes . 10559902

[46] Ono S, Miyachi Y, Arakawa A. Hair regrowth following TNF-alpha blockade in coexisting psoriasis vulgaris and alopecia areata. Eur J Dermatol. 2013;23(4):537. 10.1684/ejd.2013.2074 https://search.crossref.org/?q=Hair+regrowth+following+TNF-alpha+blockade+in+coexisting+psoriasis+vulgaris+and+alopecia+areata&from_ui=yes . 24047580

[47] Tong X, Coulombe PA. Keratin 17 modulates hair follicle cycling in a TNFalpha-dependent fashion. Genes Dev. 2006;20(10):1353–64. 10.1101/gad.1387406 https://search.crossref.org/?q=Keratin+17+modulates+hair+follicle+cycling+in+a+TNFalpha-dependent+fashion&from_ui=yes . 16702408 PMC1472909

[48] Wang X, Chen H, Tian R, Zhang Y, Drutskaya MS, Wang C, et al. Macrophages induce AKT/beta-catenin-dependent Lgr5(+) stem cell activation and hair follicle regeneration through TNF. Nat Commun. 2017;8(1):14091. 10.1038/ncomms14091 https://search.crossref.org/?q=Macrophages+induce+AKT/beta-catenin-dependent+Lgr5(+)+stem+cell+activation+and+hair+follicle+regeneration+through+TNF.+Nat+Commun&from_ui=yes . 28345588 PMC5378973

[49] Nowaczyk MJ, Thompson BA, Zeesman S, Moog U, Sanchez-Lara PA, Magoulas PL, et al. Deletion of MAP2K2/MEK2: a novel mechanism for a RASopathy? Clin Genet. 2014;85(2):138–46. 10.1111/cge.12116 https://search.crossref.org/?q=Deletion+of+MAP2K2/MEK2:+a+novel+mechanism+for+a+RASopathy?+Clin+Genet&from_ui=yes . 23379592 PMC4480871

[50] Brown CW, Houston-Hawkins DE, Woodruff TK, Matzuk MM. Insertion of Inhbb into the Inhba locus rescues the Inhba-null phenotype and reveals new activin functions. Nat Genet. 2000;25(4):453–7. 10.1038/78161 https://search.crossref.org/?q=Insertion+of+Inhbb+into+the+Inhba+locus+rescues+the+Inhba-null+phenotype+and+reveals+new+activin+functions.+Nat+Genet&from_ui=yes . 10932194

[51] Xu H, Ma G, Mu F, Ning B, Li H, Wang N. STAT3 Partly Inhibits Cell Proliferation via Direct Negative Regulation of FST Gene Expression. Front Genet. 2021;12:678667. 10.3389/fgene.2021.678667 . 34239543 PMC8259742

[52] Bertola DR, Pereira AC, de Oliveira PS, Kim CA, Krieger JE. Clinical variability in a Noonan syndrome family with a new PTPN11 gene mutation. Am J Med Genet A. 2004;130A(4):378–83. 10.1002/ajmg.a.30270 https://search.crossref.org/?q=Clinical+variability+in+a+Noonan+syndrome+family+with+a+new+PTPN11+gene+mutation.+Am+J+Med+Genet+A.+&from_ui=yes . 15384080

[53] Sayama K, Hanakawa Y, Nagai H, Shirakata Y, Dai X, Hirakawa S, et al. Transforming growth factor-beta-activated kinase 1 is essential for differentiation and the prevention of apoptosis in epidermis. J Biol Chem. 2006;281(31):22013–20. 10.1074/jbc.m601065200 https://search.crossref.org/?q=Transforming+growth+factor-beta-activated+kinase+1+is+essential+for+differentiation+and+the+prevention+of+apoptosis+in+epidermis&from_ui=yes . 16754690

[54] Sayama K, Kajiya K, Sugawara K, Sato S, Hirakawa S, Shirakata Y, et al. Inflammatory mediator TAK1 regulates hair follicle morphogenesis and anagen induction shown by using keratinocyte-specific TAK1-deficient mice. PLoS One. 2010;5(6):e11275. 10.1371/journal.pone.0011275 https://search.crossref.org/?q=Inflammatory+mediator+TAK1+regulates+hair+follicle+morphogenesis+and+anagen+induction+shown+by+using+keratinocyte-specific+TAK1-deficient+mice&from_ui=yes . 20585657 PMC2890581

[55] Yang YJ, Zhao R, He XY, Li LP, Wang KW, Zhao L, et al. A novel splicing mutation of KIT results in piebaldism and auburn hair color in a Chinese family. Biomed Res Int. 2013;2013:689756. 10.1155/2013/689756 https://search.crossref.org/?q=A+novel+splicing+mutation+of+KIT+results+in+piebaldism+and+auburn+hair+color+in+a+Chinese+family.+Biomed+Res+Int.+2013;2013:689756&from_ui=yes . 24000325 PMC3755434

[56] Kilsby AJ, Cruwys M, Kukendrajah C, Russell-Eggitt I, Raglan E, Rajput K, et al. Homozygosity for piebaldism with a proven KIT mutation resulting in depigmentation of the skin and hair, deafness, developmental delay and autism spectrum disorder. Clin Dysmorphol. 2013;22(2):64–7. 10.1097/mcd.0000000000000055 . 23399981

[57] Andl T, Ahn K, Kairo A, Chu EY, Wine-Lee L, Reddy ST, et al. Epithelial Bmpr1a regulates differentiation and proliferation in postnatal hair follicles and is essential for tooth development. Development. 2004;131(10):2257–68. 10.1242/dev.01125 https://search.crossref.org/?q=Epithelial+Bmpr1a+regulates+differentiation+and+proliferation+in+postnatal+hair+follicles+and+is+essential+for+tooth+development.+Development&from_ui=yes . 15102710

[58] Yuhki M, Yamada M, Kawano M, Iwasato T, Itohara S, Yoshida H, et al. BMPR1A signaling is necessary for hair follicle cycling and hair shaft differentiation in mice. Development. 2004;131(8):1825–33. 10.1242/dev.01079 . 15084466

