# Supplementary figures and images for: Identification of key genes and signaling pathways related to Hetian sheep wool density by RNA-seq technology
Source: PLoS One. 2022 May 25;17(5):e0265989. doi: 10.1371/journal.pone.0265989 (PMC9132291; doi:10.1371/journal.pone.0265989)

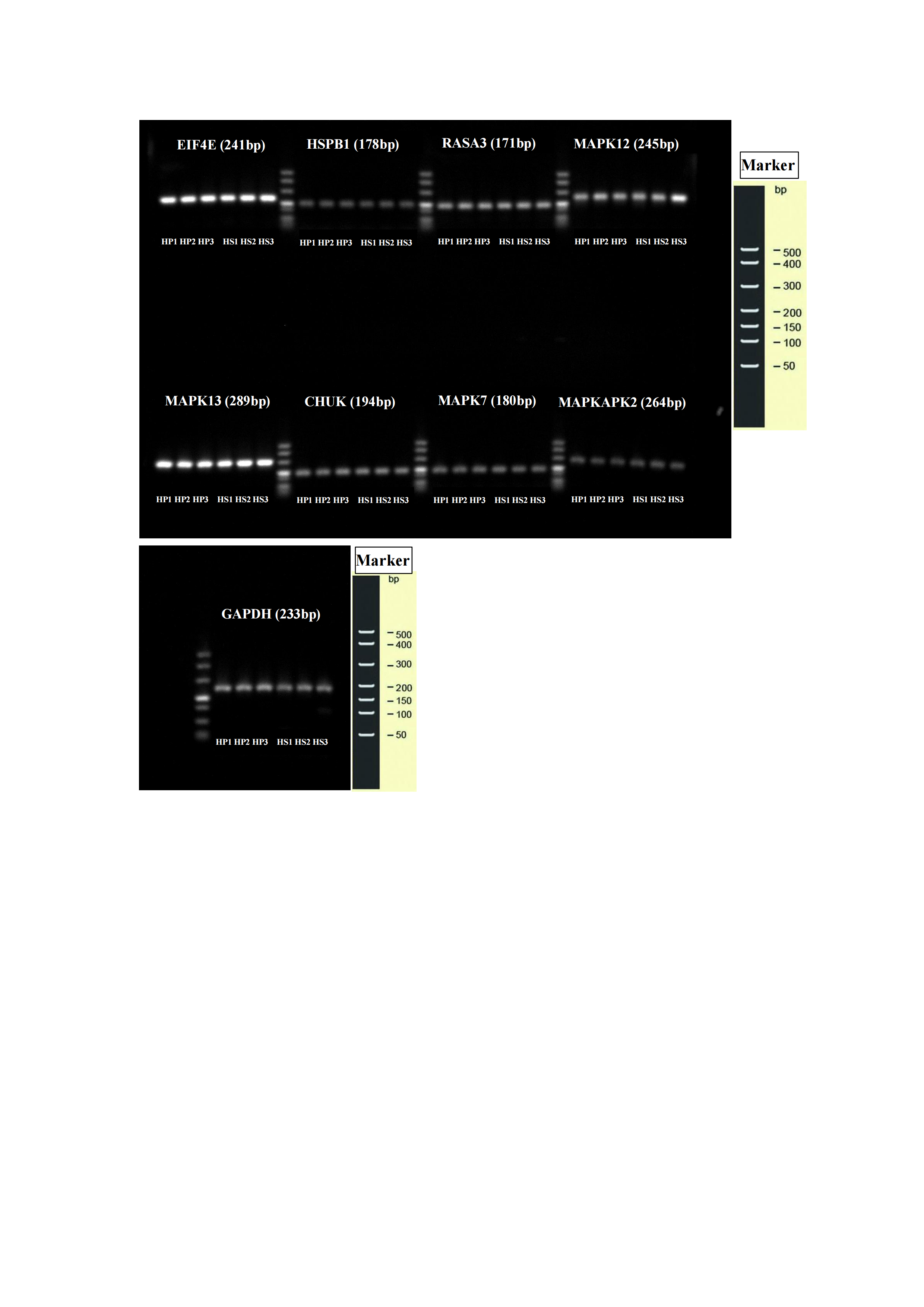

Supplement: S1 Raw images — (TIF) [file pone.0265989.s005.tif]
